# Genetic assessment of captive red panda (*Ailurus fulgens*) population

**DOI:** 10.1186/s40064-016-3437-1

**Published:** 2016-10-07

**Authors:** Arun Kumar, Upashna Rai, Bhupen Roka, Alankar K. Jha, P. Anuradha Reddy

**Affiliations:** 1 CSIR-Centre for Cellular and Molecular Biology, Uppal Road, Hyderabad, 500 007 India; 2 Padmaja Naidu Himalayan Zoological Park, Darjeeling, 734101 India

**Keywords:** Red panda, Captive breeding, Genetics, Population viability

## Abstract

Red panda *(Ailurus fulgens)* is threatened across its range by detrimental human activities and rapid habitat changes necessitating captive breeding programs in various zoos globally to save this flagship species from extinction. One of the ultimate aims of ex situ conservation is reintroduction of endangered animals into their natural habitats while maintaining 90 % of the founder genetic diversity. Advances in molecular genetics and microsatellite genotyping techniques make it possible to accurately estimate genetic diversity of captive animals of unknown ancestry. Here we assess genetic diversity of the red panda population in Padmaja Naidu Himalayan Zoological Park, Darjeeling, which plays a pivotal role in ex situ conservation of red panda in India. We generated microsatellite genotypes of fifteen red pandas with a set of fourteen loci. This population is genetically diverse with 68 % observed heterozygosity (H_O_) and mean inbreeding (F_IS_) coefficient of 0.05. However population viability analysis reveals that this population has a very low survival probability (<2 %) and will rapidly loose its genetic diversity to 37 % mainly due to small population size and skewed male-biased sex ratio. Regular supplementation with a pair of adult individuals every five years will increase survival probability and genetic diversity to 99 and 61 % respectively and will also support future harvesting of individuals for reintroduction into the wild and exchange with other zoos.

## Background

Red panda (*Ailurus fulgens*), also known as the lesser panda, is one of earth’s living fossils and its ancestors can be traced back to more than ten million years ago across Eurasia (Mayr 1986). Presently red panda populations are sporadically distributed in bamboo forests of Himalayan and Heng-Duan mountains in Nepal, India, Bhutan, Myanmar and Southwestern China (Su et al. 2001; Li et al. 2005). These populations continue to drastically decline across their habitats due to hunting, poaching, habitat loss and fragmentation (Wei et al. 1999; Choudhury 2001; Jha 2011). Red panda is classified as vulnerable by IUCN and is listed in Appendix I of CITES (Convention on International Trade in Endangered Species of Wild Flora and Fauna) since its wild population is estimated to be less than 10,000 mature individuals (Wang et al. 2008). Although endemic to eastern Himalayas and a flagship species for conservation of this fragile ecosystem, little is known about the genetic diversity of red panda in the wild, as it is shy, scansorial and dwells in altitudinal ranges of 1500–4800 m making it a very difficult animal to study (Choudhury 2001). Recent research on demography, phylogeography and genetic diversity of red panda (Su et al. 2001; Li et al. 2005; Hu et al. 2011) opens more opportunities to study current trends in wild red panda populations.

Several ex situ breeding programs have been initiated worldwide to protect this iconic species as its future survival relies on implementation of active conservation measures. Ex situ management and captive breeding for species conservation have grown enormously in recent years, and are the ultimate alternatives to in situ conservation, for preservation and recovery of endangered species aiming towards their reintroduction in the wild (Ballou 1992; Snyder et al. 1996; Ramirez et al. 2006) along with other aspects like education, research and fund raising. Founding and managing populations in captivity improve chances of long term survival of the species and can be used to supplement wild populations (Ramirez et al. 2006; Araki et al. 2007). Though widely accepted as a conservation tool, captive breeding/management of small populations comprises loss of genetic diversity, difficulty in achieving breeding, disease epidemics, adaptation to selection pressures in captivity, inbreeding and genetic drift (O’Grady et al. 2006; Zeoli et al. 2008; Ouborg et al. 2010). Genetic drift and inbreeding are known to increase homozygosity and accumulation of deleterious alleles thus decreasing both long and short term viabilities of populations by reducing the individual’s fitness and adaptive potential to changing environments (Ouborg et al. 2010). Genetic consequences of captive breeding can nevertheless be managed in pedigreed populations by preventing breeding between related individuals and by introducing new animals to conserve genetic variation and gene pool of the representative (founder) animals. Recently however molecular genetic management measures have been employed to estimate relatedness and variations for many captive populations with individuals of unknown ancestry, (Russello and Amato 2004; Walling et al. 2011).

Advances in molecular genetics and genomics and consequent use of small informative fragments of DNA, like microsatellites, can help infer levels of genetic variation, inbreeding and relatedness within a population. Microsatellites are hypervariable loci, and are popular markers for population and conservation genetic studies at the intraspecific level due to their high mutation rates (Zachos et al. 2009). Since these markers are versatile, cost effective and the results are reproducible, genotyping of individual animals by simple PCR amplification is a sensible method to estimate genetic diversity, population size and structure, migration rates, kinship and parentage of various endangered species (Hoffman and Amos 2005; Guichoux 2011). The status and efficiency of demographic and genetic management measures for a captive population can then be evaluated and graded by Population Viability Analysis or PVA (Akçakaya and Sjögren-Gulve 2000; Brook et al. 2000; Holmes et al. 2007; Greenwald 2010). PVA estimates the extinction risk and effective population size of endangered species, by modeling effects of demographic and genetic stochasticities, and influences of management measures on the endangered population (Yang et al. 2007). It is a sensitive population model, and is often applied with care for those species where the availability of demographic data is minimal (Greenwald 2010).

India has very small captive populations of approximately twenty-five red panda housed at the Padmaja Naidu Himalayan Zoological Park (PNHZP), Darjeeling, and Himalayan Zoological Park (HZP), Gangtok. These animals are periodically exchanged with international zoos as part of the Species Survival Plan to maintain genetic diversity among captive bred individuals. The two Indian zoo populations of red panda have the distinctive priority of being located within the range states of its wild population distribution; and importantly being part of international captive breeding program, have the potential to provide a link between captive and wild populations (Glatston 2011). Therefore these comparatively small captive populations play a very important role in conservation of red panda (Glatston 2011). In this study we describe the genetic diversity of captive red pandas in PNHZP, Darjeeling, with a panel of fourteen microsatellite loci and project the viability of this population to maintain founder genetic diversity in the captive-bred individuals for a period of 100 years.

## Methods

### Sampling and DNA isolation

Padmaja Naidu Himalayan Zoological Park (PNHZP), Darjeeling plays a pivotal role in red panda captive breeding in India, and had fifteen individuals, including three founders (two males and one female) and twelve captive-born at the time of our study (2012–2013). Fresh faecal samples of all fifteen red pandas (five females and ten males) were collected in sterile bottles with 90 % ethanol. Out of these animals, blood samples from four males and three females were also collected in EDTA-coated vacutainer tubes. Samples were acquired as per the Padmaja Naidu Himalayan Zoological Park and Central Zoo Authority, Govt. of India, ethical guidelines and procedures. No animal was injured as a result of this study. All samples were transported to the laboratory and stored at −20 °C until DNA isolation. Genomic DNA from blood was isolated by standard phenol–chloroform method (Sambrook et al. 1989). DNA was isolated from the external epithelial layer of fecal samples with QIAamp DNA stool kit (Qiagen, USA) as per the manufacturer’s instructions.

### Microsatellite amplification

The following loci were used for microsatellite genotyping—AF2, AF4, AF6, AF7, AF21, AF23, AF24 (Liang et al. 2007) RP4, RP5, RP6, RP8, RP9, RP11, RP12 (Wu et al. 2008) with an average reported polymorphism of five to seven alleles per locus. All forward primers were labeled at the 5′ end with either HEX or FAM fluorescent dye (Bioserve Inc, India). Briefly, a 15 µl singleplex reaction contained 200 mM of each dNTP, 10X Ex-Taq Buffer (Takara, Japan), 10× BSA, and 0.75U Ex-Taq Polymerase (Takara, Japan), 1.5 mM each of forward and reverse primers, and ~10 ng of template DNA. PCR amplifications were carried out in an epgradient S Mastercycler (Eppendorf, Germany) with initial denaturation at 94 °C for 10 min and 40 cycles of 94 °C for 30 s, annealing at Tm of each primer (Table 1) for 20 s, 72 °C for 20 s, followed by a final extension for 10 min at 72 °C. All reactions were performed for a minimum of three repeats following a multi-tube approach (Taberlet et al. 1996) and were set up in a hood cleaned with bleach and alcohol, and irradiated with UV light to eliminate PCR contaminants. A negative control was included in all PCR reactions. Post amplification, PCR products were visualized by agarose gel electrophoresis, were electrophoresed with allelic size standard LIZ 500 (Applied Biosystems, USA) in an ABI Prism 3730 genetic analyzer (Applied Biosystems, USA), and alleles were scored with GeneMapper v3.2 (Applied Biosystems, USA).

**Table 1:** Statistics on captive red panda genotypes obtained from fourteen microsatellite markers

Locus	Repeat	Range (bp)	Tm	References	k	N	H_O_	H_E_	PIC	HW	F(IS)
AF2	(CTAT)11	120–144	57 °C	Liang et al. (2007)	5	13	0.538	0.655	0.581	0.2158	0.184
AF4	(GATA)15	231–255	56 °C	Liang et al. (2007)	6	14	0.857	0.817	0.757	0.1232	−0.051
AF6	(GATA)16	205–253	54 °C	Liang et al. (2007)	5	15	0.667	0.726	0.645	0.8253	0.085
AF7	(GATA)11	213–243	60 °C	Liang et al. (2007)	5	14	0.786	0.743	0.674	0.8539	−0.059
AF23	(TATC)13	133–161	62 °C	Liang et al. (2007)	6	14	0.786	0.741	0.671	0.9241	−0.063
AF24	(TATC)10	225–243	62 °C	Liang et al. (2007)	4	11	0.727	0.675	0.586	0.7636	−0.081
RP5	(CA)23	110–130	53 °C	Wu et al. (2008)	4	15	0.667	0.671	0.586	0.7103	0.007
RP6	(CA)13	155–167	53 °C	Wu et al. (2008)	3	14	0.286	0.455	0.386	0.0445	0.381
RP11	(CA)12	105–127	54 °C	Wu et al. (2008)	6	12	0.917	0.772	0.697	0.8248	−0.198
RP12	(CA)16	131–147	52 °C	Wu et al. (2008)	3	15	0.467	0.618	0.517	0.0791	0.252
AF21	(GATA)11	176–208	58 °C	Liang et al. (2007)	7	10	0.9	0.821	0.748	0.399	−0.102
RP4	(GT)19	198–212	54 °C	Wu et al. (2008)	5	14	0.714	0.796	0.729	0.0727	0.107
RP8	(CA)28	163–173	56 °C	Wu et al. (2008)	4	11	0.455	0.662	0.587	0.0291	0.324
RP9	(CA)20	125–143	55.5 °C	Wu et al. (2008)	6	9	0.778	0.85	0.774	0.8824	0.089
							0.6818	0.7144	0.6384		0.048

*Tm* annealing temperature, *K* number of alleles per locus, *N* number of Individuals typed at each locus, *H*_*O*_ observed heterozygosity, *H*_*E*_ expected heterozygosity, *PIC* polymorphism information content, *HW* Hardy–Weinberg equilibrium

### Allele frequency analysis

Allele frequency and identity analyses were implemented as described by Reddy et al. (2012) to match genotypes of individuals from blood and faecal DNA with Identity test in CERVUS 3.0 (Kalinowski et al. 2007). Probability of identifying an unrelated individual (P_ID_) and sibling (P_SIB_) from a pair of genotypes was determined from allele frequencies, and minimum number of loci required to differentiate siblings was restricted to nine loci. Mismatched genotypes were re-examined manually for scoring errors, and in some cases the respective loci were genotyped again. All loci were scored manually for genotyping errors, amplification success and certainty of PCR repetitions in Microsoft EXCEL spreadsheets.

### Genetic diversity analysis

Consensus genotypes were used to determine overall expected (H_E_) and observed (H_O_) heterozygosities using allele frequency function in CERVUS 3.0. Polymorphism Information Content (PIC) of heterozygous loci was also obtained from allele frequencies. Alleles deviating from Hardy–Weinberg equilibrium (HWE) were tested with Genepop v4 (Rousset 2008) with default Markov chain parameters. Linkage disequilibrium (LD) estimates between loci and inbreeding coefficient (F_IS_) in the population were determined using FSTAT (Goudet 1995).

### Viability analysis

Population viability analyses of the captive red panda population were simulated with Vortex v10 (Lacy 1993, 2000). All simulations were projected for a period of 100 years with 1000 burn-ins.

#### Baseline scenario

Basic demographic parameters were used to project the baseline scenario in viability analysis. Red panda is a polygynous mammal and generally both sexes mature at 18–24 months and reproduce till 12 years of age. These animals brood once a year and have 1–4 offspring per brood with a 1:1 sex ratio. Broods with 1 or 2 cubs are more frequent, and litters with 4 offspring are very rare (Glatston 2006). The captive population described in this study is skewed and consists of five females and ten males in reproductively active age. Default inbreeding value of 3.14 lethals per diploid individual was considered as per the mean values estimated from 40 different captive species of mammals (O’Grady et al. 2006). Red pandas in captivity exhibit high mortality rates till 2 years of age. All simulations were projected with default scenarios along with genetic management without supplementing to/harvesting from the existing population. A file on allele frequencies calculated from microsatellite data was added under genetic management in Vortex *v*10. Carrying capacity of PNHZP was predicted to be a maximum of 50 red pandas for future increase of population.

#### Supplementation scenario

In order to maintain 90 % of the genetic diversity present in founder/wild population for a period of 100 years, captive populations need supplementation on a timely basis either from the wild or from other zoos. Although an ideal scenario requires supplementation with two females and one male once every year or 2 years, the practical reality is that only one animal on an average can be acquired every 2 or 3 years due to logistics problems and difficulties in obtaining permits. Therefore simulations were projected by supplementing one adult male and one adult female once in 5 years until 100th year.

#### Supplementation and harvesting scenario

PNHZP has an important role to play in captive breeding of red panda especially since it is located in a range state of its wild population distribution. This adds a new dimension and responsibility on PNHZP to provide wild-caught animals to international zoos to infuse wild blood into their stocks and also to release captive-bred animals to replenish dwindling wild populations. Simulations were projected by harvesting one adult male and one adult female once in 5 years till 100th year while supplementing individuals as mentioned above.

#### Scenarios with catastrophes

Although protected and cared for in captivity, there can be several local or widespread catastrophes impeding an animal’s growth. So far the red panda population at PNHZP has not been affected by any catastrophic event resulting in major loss of population. However we cannot rule out future possibilities especially since Darjeeling has high incidences of landslides, heavy rainfall, etc., and there is always a chance for outbreaks of viral diseases like canine distemper. Effects of catastrophes were included in baseline scenario as well as supplementation and harvesting scenarios, and these were projected to analyze the probability of population extinction at each incidence of catastrophic events. Although landslides and heavy rainfall have not majorly affected reproductive and survival efficiencies of PNHZP red panda population so far, we projected a reduction in reproductive efficiency by 3 % and survival efficiency by 1 % during these events. Disease outbreak can be a serious phenomenon that can wipe out the entire population. However the frequency or probability of occurrence of an outbreak is less and may happen once in 10 years in contrast to environmental catastrophes mentioned above which can occur every year.

## Results

### Microsatellite statistics

We genotyped all fifteen red pandas at PNHZP at a minimum of eleven microsatellite loci, and got an average dropout rate of 9.8 % at fourteen loci and 89 % amplification success rate (Table 2). We needed an average of four PCR repetitions to obtain genotypes with 99 % certainty at each locus (data not shown). Probability of identifying an individual (P_ID_) from a pair of unrelated individuals is 0.0001 with a minimum of six least variable loci and 9.7 × 10^−13^ with all fourteen loci, while probability of identifying siblings (P_SIB_) from pair of animals is 0.001 with nine least variable loci and 1.06 × 10^−5^ with all fourteen loci. Identity analysis in CERVUS 3.0 correctly matched genotypes obtained from faecal and blood DNA samples of each animal. Genotypes obtained with a minimum of eleven loci were sufficient to generate genetic diversity parameters effectively.

**Table 2:** Locus-wise PCR success and allelic dropout rates, and number of PCR repeats required to obtain genotypes with 99 % certainty

Locus	PCR success (%)	Dropout rate (%)	Certainty of repeats
AF2	92.68	9.84	4
AF4	88.51	5.97	3
AF6	94.95	6.45	3
AF7	87.78	11.48	4
AF21	60.26	4.55	3
AF23	88.51	8.20	3
AF24	84.44	18.42	4
RP4	96.15	1.79	3
RP5	97.22	0.00	2
RP6	82.35	9.38	4
RP8	94.44	10.34	4
RP9	89.33	15.22	4
RP11	95.24	16.22	4
RP12	92.22	19.44	4
Mean	88.86	9.81	

### Genetic diversity analysis

All fourteen microsatellites were shown to be polymorphic by Allele frequency analysis in CERVUS 3.0 with mean polymorphism information content (PIC) of 0.64, and 3–7 alleles per locus (Table 1). Expected (H_E_) and Observed (H_O_) heterozygosities were 0.714 and 0.681 respectively (Table 1). Genepop v4 (Rousset 2008) analysis with default Markov chain parameters showed that all the loci were in Hardy–Weinberg equilibrium (p < 0.001) (Table 1). Inbreeding and linkage analysis with FSTAT (Goudet 1995) indicated that none of the loci are linked to each other and the present PNHZP red panda population has a mean inbreeding value of 0.048 (Table 1).

### Population viability analysis

**Fig. 1:**
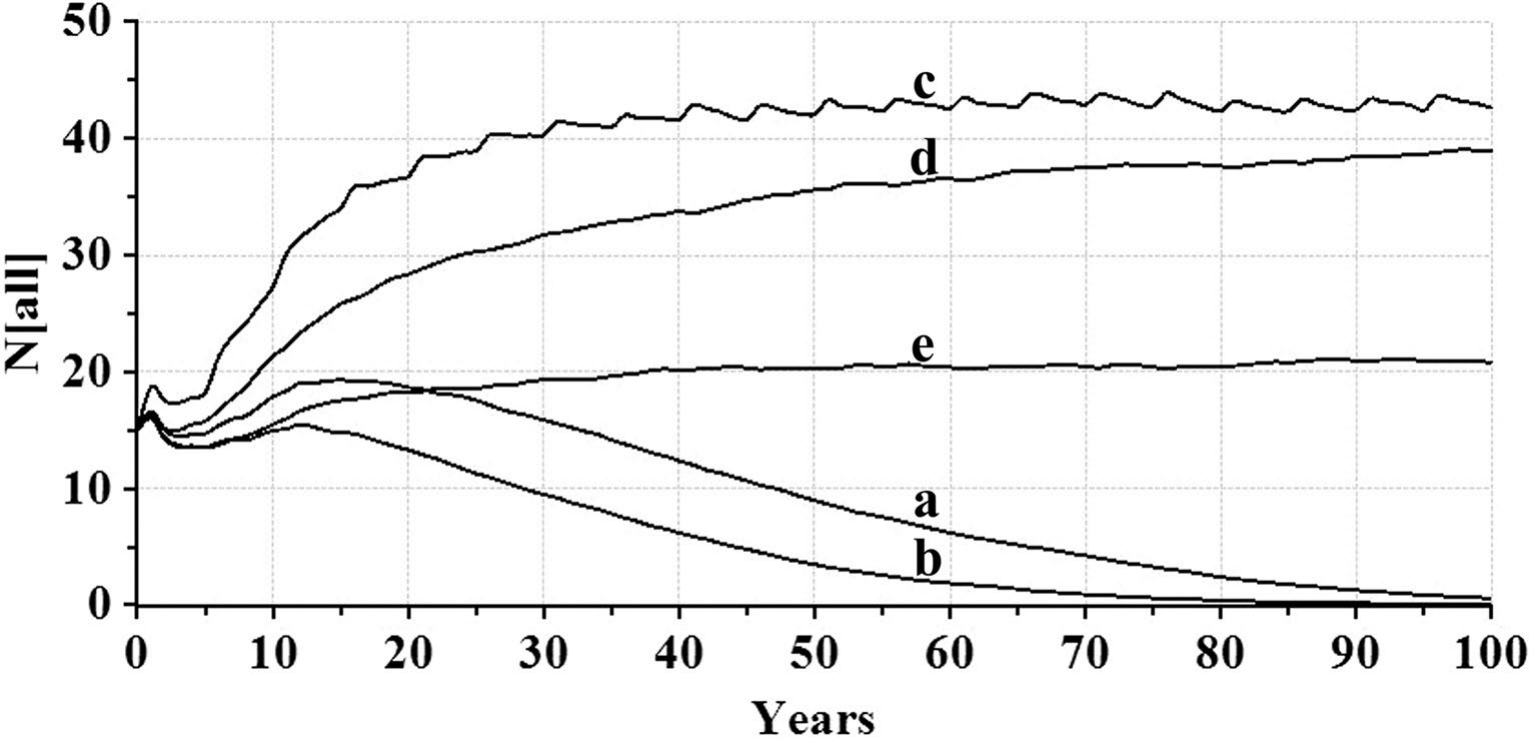
PVA graph indicating projected number of individuals (N) corresponding to simulated scenario for a period of 100 years. Scenarios are **a** baseline; **b** baseline with catastrophes; **c** supplementation; **d** supplementation and harvesting; **e** supplementation and harvesting with catastrophes

According to baseline scenario simulations, the captive red panda population at PNHZP is a slow growing population. Deterministic growth rate (assuming no stochastic fluctuations, no inbreeding depression, no supplementation and no harvest) is 10.3 % (r = 0.103, λ = 1.109), with a mean generation time of 4.27 years for both females and males, while retaining only 37 % gene diversity after 100 years. Survival probability of this population without any additional intervention is less than 2 %. Gene diversity can however be maintained at 61 % (nearly 90 % of the initial diversity of 68 %) by supplementing this population with one reproductively active female and male once every 5 years till 100th year. Supplementation also helps in increasing the population size to about 45 individuals in 25 years (Fig. 1). Harvesting individuals by translocation them to other populations or releasing them into wild along with supplementing new individuals reduces the risk of mating related individuals. Removing one female and one male from the population every 5 years in conjunction with supplementation retains the gene diversity at 61 % for 100 years. However the final number of animals is less than 40 and never reaches the full carrying capacity of PNHZP. The present red panda population is very vulnerable to environmental stochasticities and even a slight reduction in reproductive efficiency by 3 % and survival efficiency by 1 % in the baseline scenario reduces the rate of population growth to 8.6 % (r = 0.086 and λ = 1.091). When effects of these environmental factors are added to a population with supplementation and harvesting, the growth rate slows down to 7.0 % and the total number of animals never go beyond 25 individuals (Fig. 1).

## Discussion

This study attempts to understand genetic representation and population viability of captive red pandas in PNHZP, Darjeeling. We generated genetic profile of fifteen red pandas by genotyping DNA isolated from both blood and faecal samples at fourteen microsatellite loci. Although PCR reactions were performed in singleplex, these microsatellite primers show overall PCR success (88.9 %) and dropout (9.81 %) rates comparable with multiplex protocols described earlier (Morin et al. 2001; Piggott 2004; Arandjelovic et al. 2009). Observed heterozygosity (HO) level of 0.68 (Table 1) indicates that the captive red panda population at PNHZP has a genetic variation in the range of wild red panda populations in Singhalila National Park (HO = 0.63) and Neora Valley National Park (HO = 0.75) screened with the same set of microsatellite markers (data not shown).

PNHZP is pivotal in ex situ conservation of red panda in India as it is located within the country’s red panda distribution range, and is also the main Indian zoo participating in red panda Species Survival Plan. PNHZP also holds the record of successfully reintroducing captive-bred red panda in Singalila National Park in 2004 (Jha 2011). Presently the PNHZP red panda population is small and therefore extremely susceptible to demographic stochasticities. This population is also highly skewed and male-biased which predilects it to further instabilities and extinction risk. Viability analysis indicates that this is a slow growing population (r = 0.103, λ = 1.109), with a mean generation time of 4.27 years for both females and males, and will retain only 37 % of its gene diversity after 100 years. Extinction probability of this population is nearly 98 % and active measures are needed for its growth and to maintain its present genetic diversity. Although supplementation simulations show that addition of three individuals (two females and one male) will help in improving and maintaining genetic diversity close to the original value (data not shown), and also in rapidly attaining population growth, this is not practically possible. Obtaining animals either from the wild or from other zoos entails several logistic problems like permits, finances, disease surveillance, accidents during transit, etc. and ultimately the animal’s ability to adapt to and reproduce in its new environment. A more conservative estimate which is also practiced presently is addition of a pair of animals, every 5 years. This will help in retaining gene diversity at 61 % and in increasing the present population to about 40 individuals within 25 years (Fig. 1).

As PNHZP is located within a range state it becomes relatively easy for it to build up genetically diverse stock population from rescued wild red pandas which can later be used for reintroduction into the wild or can be exchanged with other zoos/subpopulations (Leus 2011). While precarious now due to skewed sex ratio and small population size, harvesting from PNHZP population should be feasible after increasing the number of breeding females. Even in its present condition and with regular supplementation, removing one female and one male from the population every 5 years will retain gene diversity at 61 % for 100 years. However, unless the supplementation rate is increased, the population size will always remain small and never quite reach the full carrying capacity of PNHZP (Fig. 1). This situation can rapidly deteriorate in cases of local environmental catastrophes like landslides, and in disease outbreaks.

The Central Zoo Authority of India proposed a Conservation Breeding Initiative in 2005 wherein zoos would actively participate in captive breeding of highly threatened species with less than 2500 individuals left in the wild, especially species with localised distributions (Sharma et al. 2008). At least 250 properly bred and physically, genetically and behaviourally healthy individuals of each target species should be managed in captivity globally, of which at least 100 individuals should be managed within India, to act as insurance in case of loss of the species in wild (Leus 2011). The proposed target of 100 red pandas managed in Indian zoos will be possible with involvement and active participation of Himalayan Zoological Park (HZP), Gangtok and Bharat Ratna Pt. Govind Ballabh Pant High Altitude Zoo, Nainital, and by regularly adding new founders from the wild.

## Conclusions

Padmaja Naidu Himalayan Zoological Park (PNHZP), Darjeeling has the largest captive population of red panda in India and is principally responsible for conservation breeding of this species in India. Although supported by two other satellite zoos in Gangtok and Nainital, their respectively populations are presently too small to influence the breeding program. Being located in the species’ range states, Darjeeling and Gangtok’s captive red panda populations have tremendous potential to grow, maintain the genetic diversity of the international captive population and restock dwindling wild populations.
